# Relationship between peri-implant soft tissue characteristics and peri-implant health and mucositis: a cross-sectional study

**DOI:** 10.1007/s44445-026-00250-z

**Published:** 2026-10-07

**Authors:** Tugba Sahin, Nazurah binti Nik Eezammuddeen

**Affiliations:** 1 Faculty of Dentistry, Division of Periodontology, Bolu Abant İzzet Baysal University, Bolu, Turkey; 2 Faculty of Dentistry, Division of Periodontology, Universiti Teknologi MARA, Shah Alam, Malaysia

**Keywords:** Dental implants, Gingival phenotype, Keratinized mucosa, Soft tissue, Peri-implant mucositis

## Abstract

This study aimed to evaluate the effects of keratinized tissue width, gingival thickness, and phenotype on peri-implant tissue health and to investigate their association with peri-implant mucositis. A total of 316 implants (165 individuals) were included in the study. Clinical and periodontal parameters, including plaque and gingival indices, probing pocket depth, bleeding on probing, gingival recession, and clinical attachment level, were recorded. The keratinized tissue width, gingival thickness, and gingival phenotype were assessed and compared between the peri-implant health and peri-implant mucositis groups. Binary logistic regression analysis was performed to identify predictors of peri-implant mucositis and gingival characteristics. The keratinized tissue width did not significantly differ between the peri-implant health and mucositis groups (p > .05). The gingival thickness and phenotype were significantly different (p < .05 and p > .05, respectively), but the effect size for gingival thickness was small (Cohen’s d = 0.27), indicating limited clinical importance. Logistic regression showed that plaque, gingival indices, gingival thickness, and gingival phenotype predicted peri-implant mucositis. Increased gingival thickness, plaque, and gingival indices were associated with a higher risk of mucositis, whereas a thin gingival phenotype was associated with a reduced risk. Attachment level was the only significant predictor of keratinized tissue width > 2 mm, whereas no variables significantly predicted gingival thickness. Although gingival thickness and phenotype were statistically associated with peri-implant mucositis, their clinical contribution was limited. Plaque accumulation and gingival inflammation remained the strongest predictors of peri-implant disease.

**Supplementary Information:**

The online version contains supplementary material available at https://doi.org/10.1007/s44445-026-00250-z.

## Introduction

Dental implants are considered the gold standard for replacing missing teeth (AlOtaibi et al. 2021). Peri-implant tissue disease can jeopardize implant longevity. In the absence of bone loss, peri-implant mucositis refers to inflammation of the mucosa surrounding endosseous tissue (Mattheos et al. 2012), (Heitz-Mayfield and Salvi 2018). Peri-implant health is characterized by the absence of erythema, bleeding on probing, swelling, and suppuration, and is synonymous with implant success (Berglundh et al. 2018), (Cortellini et al. 2019). According to Lang & Loe, teeth with at least 1 mm of attached gingiva and at least 2 mm of keratinized gingiva (KG) have improved periodontal health (Lang and Löe 1972). Plaque control is considered easier when the keratinized tissue width (KTW) exceeds 2 mm (Giannobile, Jung, and Schwarz 2018). An adequate KTW surrounding implants is associated with reduced plaque accumulation and lower levels of mucosal inflammation (Boynueğri et al. 2013). A KTW of less than 2 mm has been suggested as a potential risk factor in periodontally healthy, non-smoking (Grischke et al. 2019). By contrast, some studies have shown that the presence or absence of KG does not affect the periodontal indices used to assess periodontal health (Shimomoto et al. 2021), (Kim et al. 2009). The literature contains limited information regarding the relationships between peri-implant gingival thickness (GT) and periodontal indices and periodontal health.

Although some studies have investigated the soft tissue characteristics around dental implants separately, evidence concerning the simultaneous evaluation of KTW, GT, and gingival phenotype (GP) together is still lacking. A comprehensive evaluation of these parameters may increase the knowledge of their correlation with peri-implant health status and could assist in a more accurate clinical evaluation and risk assessment. Hence, the present study hypothesized that peri-implant health status would be associated with gingival characteristics, with peri-implant health status (peri-implant health or peri-implant mucositis) as the primary outcome and clinical parameters, including plaque index (PI), gingival index (GI), bleeding on probing (BOP), probing depth (PD), gingival recession (GR) and clinical attachment level (CAL) as secondary outcomes; specifically, insufficient KTW, thinner GT, and a thin GP were expected to be associated with peri-implant mucositis and less favorable clinical parameters. This study aims to examine the association between the KTW, GP, and GT and peri-implant tissue health and to evaluate the relationship between peri-implant health and peri-implant mucositis.

## Materials and methods

After providing information about the study’s objectives and the Declaration of Helsinki (Emanuel 2013). Principles were received, and the participants provided signed informed consent. STROBE cross-sectional study compliance information was also provided. Participants were included by a convenience sampling technique from patients visiting the Department of Periodontology at the Faculty of Dentistry, Bolu Abant İzzet Baysal University, from June 11, 2024, to April 12, 2026.

Anamnesis was obtained from implant patients who applied to Bolu Abant İzzet Baysal University. A total of 316 implants (165 individuals) were included in the study. Individuals with implants who met the inclusion criteria were included in the study. The PI (Silness and Löe 1964), GI (Loe and Silness 1963), BOP (Ainamo and Bay 1975), PD, CAL, GR, GP, GT, and KTW were measured in all implant patients. The obtained indices and radiographs were used to determine whether the implants exhibited peri-implant mucositis or peri-implant health. The patients were divided into two groups: peri-implant health (158 implants) and peri-implant mucositis (158 implants). The associations between all parameters and periodontal health were examined.

### Indices

A scale from zero to three was used to evaluate the GI and PI. Following probing, the presence or absence of bleeding was evaluated for every dental implant. The distance between the edge of the gingival margin and the bottom of the gingival sulcus is known as the PD. CAL was measured as the distance from the cementoenamel junction to the base of the gingival sulcus. Periodontal indices were measured at six sites on each tooth- mesiobuccal, mid-buccal, distobuccal, mesiolingual, mid-lingual, and distolingual-using a manual UNC-15 periodontal probe (PCP15; Hu-Friedy, Chicago, IL, USA).

A topical anesthetic agent was used to anesthetize the gingiva. A rubber stop/caliper was used to introduce an endodontic file of size 06 at a location centered between the mucogingival junction and the gingival margin. The KTW was measured using a periodontal probe to determine the distance from the gingival margin to the mucogingival line. GT was measured by inserting the periodontal probe into the sulcus and observing its reflection in the gingival tissue. If the probe was visible when placed in the sulcus, gingiva was classified as thin (≤ 1 mm), and if the reflection of the probe was not visible, it was classified as thick (> 1 mm). Clinical oral examination was performed by a single calibrated examiner [T.Ş.].

### Inclusion and exclusion criteria

Patients aged 18–65 years who were systemically healthy or had a controlled medical condition and had fixed prostheses on the implants for at least 1 year following functional prosthetic loading of the dental implant were included in the study.

Individuals with bruxism, those who smoked, were pregnant, or were breastfeeding; those who used anti-inflammatory drugs, immunosuppressants, or medications affecting the mucosa and bone; those who had undergone peri-implant disease treatment after implant placement; and those with clinically evident residual cement or those that were not correctly positioned were excluded from the study.

### Sample size and statistics

According to the results of the power analysis performed using the GPower program (GPower 3.1 software; Heinrich Heine University, Düsseldorf, Germany), for the t-test (means: difference between two independent means [two groups]), with an alpha error probability (α) of 0.04, an effect size (d) of 0.5, and a power (1 − β) of 0.95, a minimum total sample size of 272 was determined, with 136 samples required for each group.

Statistical analyses were performed using SPSS 27.0. First, the normality of the distribution was assessed using the Kolmogorov-Smirnov test. Normality of distribution for continuous clinical variables was evaluated using the Kolmogorov–Smirnov test, the Shapiro–Wilk test, as well as coefficients of skewness and kurtosis (Supplementary Table 1). Formal goodness-of-fit tests revealed statistically significant deviations from strict normal distributions across all variables (*p* < .001), which is expected given the high statistical power and sensitivity of formal tests in larger sample sizes (*N* = 316). However, for the primary continuous clinical predictors skewness and kurtosis values fell comfortably within the acceptable threshold bounds of -2.0, + 2.0. In accordance with the Central Limit Theorem (*N* = 316 > 30), the distribution shapes were deemed sufficiently robust to satisfy parametric assumptions, indicating that the data were normally distributed; therefore, it was deemed appropriate to conduct parametric tests. A total of 316 implants from 165 patients were examined in this study. To perform intergroup comparisons, the independent-samples t-test was used to analyze continuous data. To compare categorical data, the chi-square test was used. Effect sizes were calculated for significant results. Cohen’s d allows for meaningful comparisons of clinical effect magnitude across variables and independent studies regardless of sample size. The Phi coefficient was selected as the non-parametric effect size metric for the categorical variable. To identify the index values with the greatest effect on the distinction between the peri-implant health and peri-implant mucositis groups, binary logistic regression was employed. Throughout the study, statistical significance was set at *p* < .05. Multivariate regression analyses (Generalized Estimating Equations -GEE binary logistic regression) were conducted using a simultaneous forced-entry method (Enter method), in which all predetermined clinical predictor variables (PI, GI, PD, BOP, GR, CAL, GT, KTW, and GP) were entered into the model concurrently. Automated variable selection techniques—such as forward selection, backward elimination, or stepwise regression—were deliberately avoided to eliminate data-driven selection bias, control for known clinical confounders, and obtain fully adjusted parameter estimates (B, OR, and 95% CI) across all evaluated clinical variables.

## Results

### Demographic characteristics

Demographic data are presented in Table 1.

**Table 1.** Demographic Characteristics of the Participants

		f	%
Gender	Male	70	42.4
	Female	95	57.6
Education status	Primary school	40	24.2
	Elementary school	12	7.3
	High school	53	32.1
	Associate Degree	1	0.6
	University	58	35.2
	PhD	1	0.6
Working status	Present	81	49.1
	Absent	84	50.9
Systematic disease	Present	46	27.9
	Absent	119	72.1
Medicine usage	Present	32	19.4
	Absent	133	80.6
Knowledge of the dental implant brand	Present	20	12.1
	Absent	145	87.9
		**Mean ± S.D.**	**Min-Max**
Age		45.98 ± 11.83	20–86
Implant Duration		4.21 ± 2.49	1–20

### Comparisons across groups

No statistically significant difference in KTW values was observed between the peri-implant health and mucositis groups (t = 0.135, df = 314, *p*> .05, mean difference = 0.03, 95% CI= -0.45, 0.51). A statistically significant difference in the GT values was observed between the peri-implant health and mucositis groups (t=-2.320, df = 314, *p*< .05, mean difference = -0.15, 95% CI= -0.27, -0.03). The GT value was greater in the peri-implant mucositis group. On the basis of the effect size obtained, GT had a statistically significant (t=-2.320, df = 314, *p*< .05, mean difference = -0.15, 95% CI= -0.27, -0.03) but clinically small effect between groups (Cohen’s d= 0.27). When the peri-implant health and mucositis groups were compared on the basis of GP, a statistically significant difference was observed with a medium effect (x^2^ = 3.362, df = 1, *p*< .05, mean difference = 10.2%, 95% CI= -0.6, 21, Phi = 0.182). A greater proportion of thin GP was observed in the peri-implant health group than in the peri-implant mucositis group. The mean age of the study participants was 45.98 .±11.83 years, spanning a broad adult range from 20 to 86 years. The average implant duration was 4.21 ± 2.49 years, ranging from 1 to 20 years across observations (Table 2).

**Table 2:** Comparison of Groups According to KTW, GT, and GP

		*N*	Mean	S.d.	Mean Difference (95% CI L, U)	t	df	*p*	Cohen’s d
KTW	Peri-implant Health	157	2.21	2.24	0.03 (-0.45, 0.51)	0.135	314	0.893	0.01
	Peri-implant Mucositis	158	2.18	2.11					
GT	Peri-implant Health	158	1.35	0.51	-0.15 (-0.27, -0.03)	-2.32	314	0.021*	0.27
	Peri-implant Mucositis	158	1.5	0.61					
		**Thin**	**Thick**	**Total**		**x** ^**2**^	**df**	**p**	**Phi**
GP	Peri-implant Health	102 (64.6%)	56 (35.4%)	158 (100.0%)	10.2% (-0.6%, 21.0%)	3.362	1	0.043*	0.182
	Peri-implant Mucositis	86 (54.4%)	72 (45.6%)	158 (100.0%)					

Keratinized tissue width: KTW, Gingival thickness: GT, Gingiva phenotype: GP, *p< .05

### Regression analysis

The General Estimation Equations Binary logistic regression analysis showed that the model was statistically significant (χ² = 436.680, df = 8, *p* < .05). PI, GI, and GT were significant predictors of peri-implant mucositis. An increase in the PI significantly increased the risk of peri-implant mucositis (B = 1.038, *p* < .05, OR = 2.823, 95% CI: 1.222–6.526). For the PI, the unscaled Odds Ratio reflects a full 1.0-unit increase. For clinically relevant interpretation across fractional changes, each 0.1-unit increment in PI corresponds to an adjusted Odds Ratio of 1.109 (95% CI: 1.020–1.206, calculated as e^0.1×B^). An increase in GI was strongly associated with peri-implant mucositis (B = 9.745, *p*<.001). Because the observed GI values strictly ranged from 0.0 to 1.0, the unscaled Odds Ratio reflects a theoretical shift across the entire scale (OR = 17068.67). When evaluated per 0.1-unit clinical increment, each 0.1-unit increase in GI corresponded to a 2.65-fold increase in the likelihood of mucositis (OR_0.1_ =2.650, *p* < .05, 95% CI: 2.123–3.308). GT was also a significant predictor, with each 1-unit increase in GT associated with a 1.60-fold increase in the likelihood of mucositis (B = 0.470, *p* < .05, OR = 1.600, 95% CI: 1.034–2.477) (Table 3). Other variables, including the GP, PD, BOP, GR, CAL, and KTW, were not significantly associated with peri-implant health. The model constant was − 4.006 (*p* > .05).

**Table 3:** Binary Logistic GEE Regression Results – Peri-implant Health Status

	B	S.E.	Wald	df	*p*	Odds Ratio	95% C.I.for Odds Ratio		VIF
							Lower	Upper	
PI	1.038	0.427	5.897	1	0.015*†	2.823	1.222	6.526	1.025
GI	9.745	1.132	74.074	1	0.001*†	17068.67	1856.19	156955.88	3.947
PD	0.029	0.323	0.008	1	0.928	1.03	0.547	1.939	1.53
BOP (%)	2.07	117.708	0	1	0.986	0.925	0	1.241	4.956
GR	-0.563	0.596	0.892	1	0.345	0.569	0.177	1.832	1.2999
CAL	-0.196	0.283	0.48	1	0.488	0.822	0.472	1.431	1.729
GT	0.47	0.223	4.443	1	0.035*	1.600	1.034	2.477	4.174
KTW	-0.007	0.057	0.015	1	0.902	0.993	0.889	1.109	1.042
GP- Thin	-0.422	0.251	2.821	1	0.093	0.656	0.401	1.073	3.051
Constant	-4.006	2.583	2.405	1	0.092	0.018			

Plaque index: PI, Gingival index: GI, Probing depth: PD, Bleeding on probing: BOP, Gingival recession: GR, Clinical attachment level: CAL, Gingival thickness: GT, Keratinized tissue width: KTW, Gingival fenotype: GP

Overall model goodness-of-fit was confirmed via the Corrected Quasi-Likelihood under Independence Model Criterion (QICC = 20.00 for the full predictor model vs. 440.37 for the null model), indicating substantial improvement over the intercept-only baseline model

*p < .05, Cox & Snell: 0.750, Nagelkerke: 0.794, Hosmer Lemeshow Test: x^2^: 1.042, df:8, p:0.997, Model Test: x^2^: 436.680, df:8, p:0.001, AUC: 0.993(95% CI (0.9822–0.999)) Reference category = Peri-implant Health

† Due to the narrow observed ranges of clinical indices and to facilitate meaningful clinical interpretation, unscaled Odds Ratios represent a full 1.0-unit increase, whereas fractional changes reflect more practical shifts: for the GI, the 1.0-unit transition (B = 9.745, OR = 17,068.67, 95% CI:1856.19–156,955.88) corresponds to an adjusted Odds Ratio of 2.650 (95% CI: 2.123–3.308) per 0.1-unit increment; similarly, for the PI, the 1.0-unit increase (B = 1.038, OR = 2.823, 95% CI: 1.222–6.526) corresponds to an adjusted Odds Ratio of 1.109(95%” CI”:1.020–1.206) per 0.1-unit increment (calculated as e^0.1×B^ )

Overall model goodness-of-fit was confirmed via the Corrected Quasi-Likelihood under Independence Model Criterion (QICC = 20.00 for the full predictor model vs. 440.37 for the null model), indicating substantial improvement over the intercept-only baseline model. To evaluate model adequacy and predictive accuracy, diagnostic assessments were conducted for the binary logistic regression model. The Hosmer–Lemeshow goodness-of-fit test revealed no significant difference between observed and predicted values (x^2^ = 1.042, df = 8, *p* = .998), indicating excellent model fit. Variance Inflation Factor (VIF) values ranged from 1.025 to 4.956, confirming the absence of severe multicollinearity among the predictors.

The model demonstrated strong classification accuracy (95.89%), with a sensitivity of 97.47% and a specificity of 94.30% at a 0.50 cutoff value. Receiver Operating Characteristic (ROC) curve analysis confirmed outstanding discrimination power, yielding an AUC of 0.9937 (95% CI: 0.9822–0.9992, *p* = .001). Residual diagnostics further supported model stability across observations.

## Discussion

The hypothesis tested was that KTW, GT, and GP influence peri-implant tissue health and the risk of peri-implant mucositis. The results revealed that the KTW values did not differ significantly between the peri-implant health and mucositis groups, whereas GT and GP values exhibited statistically significant differences, although the effect size for GT was clinically small. Increased GT and higher PI and GI values were associated with an elevated risk of peri-implant mucositis, whereas a thin GP appeared to have a protective effect. Based on these findings, our data suggest that multiple interacting factors influence peri-implant tissue health and that gingival characteristics alone may not be sufficient to predict peri-implant disease.

A systematic review of seven studies examined KG and peri-implant health. KG reduces mucosal inflammation, plaque formation, peri-implant instability, and mucosal recession, which can contribute to implant loss (Brito et al. 2014). In another study, no statistically significant differences were observed in the modified plaque index (mPI), modified bleeding index, or PD between groups with or without KG, indicating a substantially greater effect size than that observed in the present study. These findings suggest that the presence of KG may reduce the risk of bone resorption and help maintain peri-implant tissue health (Shimomoto et al. 2021). In patients who maintained good oral hygiene and received regular implant maintenance therapy, implants with < 2 mm of peri-implant KG were more prone to lingual plaque accumulation and bleeding, as well as buccal soft tissue recession over a 5-year follow-up period. Thus, lingual areas with minimal KG should be monitored throughout maintenance therapy for early inflammatory alterations (Schrott et al. 2009). According to Souza et al. (2015), implant locations with a KTW of < 2 mm showed higher levels of inflammation, plaque buildup, and brushing pain. These results suggest that inadequate keratinized mucosa may adversely affect oral hygiene performance and increase the likelihood of peri-implant disease. Our multivariable model’s substantial correlations between PI (OR = 2.823) and GI (OR = 1.706) imply that biofilm-related parameters may have a bigger impact on peri-implant mucositis than KTW alone (Souza et al. 2016). In a study including 412 implants, Kajorn et al. (2020) reported that the nonkeratinized mucosa group exhibited greater plaque accumulation, increased mucosal recession, PD ≥ 3 mm, and greater interproximal bone level loss (Kungsadalpipob et al. 2020). In a study of 967 dental implants across 211 patients with up to 15 years of follow-up, the modified approximal PI, modified sulcus bleeding index, and BOP values were significantly greater in the group lacking KG. No significant differences were observed in the PD or vertical bone level between the groups. KG improves peri-implant tissue health but not bone level (Ladwein et al. 2015). Compared with implants with a KTW ≥ 2 mm, implants with a KTW < 2 mm did not show increased marginal bone loss or GR. A peri-implant KTW < 2 mm has been associated with increased modified PI and greater discomfort after toothbrushing (Ravidà et al. 2022). Our findings support the comprehensive study and meta-analysis by Ravidà et al., who concluded that the overall effect of insufficient KGW on peri-implant disease is limited. The likelihood of a KTW ≥ 2 mm decreased with increasing attachment loss. From a clinical perspective, increased attachment loss may compromise the stability of marginal tissues, thereby reducing the KTW. The lack of significant associations between GT and the investigated clinical parameters and periodontal indices suggests that GT may be influenced by factors beyond routine periodontal indices. In our GEE binary logistic regression model, KTW was not a significant predictor for peri-implant mucositis. GT was, however, an independent predictor. Otherwise, a statistically significant result does not necessarily mean it is clinically significant.

A keratinized mucosal band of a certain width is not necessary for preserving peri-implant tissue; however, the absence of sufficient KG around the implant may complicate proper oral hygiene practices and negatively affect aesthetic outcomes (Chiu et al. 2015). The presence of KG around the implant may not be decisive in clinical situations where adequate plaque control is ensured. Although a strong relationship between the presence of KG and peri-implant health has not been demonstrated, utmost care should be taken to preserve existing KG during treatment procedures. KTW was not found to be a significant predictor of peri-implant mucositis in the GEE binary logistic regression model. Evidence indicating that grafting procedures aimed at increasing the amount of KG improve implant treatment outcomes is limited (Wennström and Derks 2012). Kim et al. (2009) reported no significant variations in the GI, PI, or PD, with or without KG. The presence of insufficient KG around the implant may not affect hygiene or soft tissue health (Kim et al. 2009). In contrast to our results, a recent systematic review found that soft-tissue augmentation procedures that increased GT and KG were associated with improved peri-implant clinical outcomes, including reduced PD and PI, and greater marginal bone level stability. The differences between the present findings and the systematic review may be explained by differences in study design (Tavelli et al. 2021). The clinical significance of the peri-implant mucosal biotype was further emphasized in a recent comprehensive analysis establishing its association with peri-implant health, peri-implantitis severity, plaque formation, BOP, crestal bone alterations, and esthetic results. These findings provide support for the concept that the characteristics of the peri-implant soft tissue should be considered in treatment planning. However, the heterogeneity of the included studies suggests that differences in the assessment of the phenotype, study design, and definitions of outcomes may be responsible for conflicting findings for individual soft tissue parameters (Veena and Shetty 2026). The lack of significant associations between KTW and the evaluated clinical parameters (PI, GI, PD, BOP, and GR) suggests that these routinely used periodontal indicators may have limited predictive value for KG dimensions. The absence of a significant association among the clinical parameters assessed using KTW suggests that the width of keratinized tissue may not be an independent predictor of peri-implant mucositis and is not sufficient on its own to explain peri-implant health status.

Monje et al. (2023) reported that insufficient KTW (< 2 mm) is associated with local discomfort during oral hygiene procedures and is linked to an increased risk of peri-implant disease development; the lack of a significant association for KTW suggests that soft tissue characteristics alone have limited predictive value for peri-implant mucositis (Monje, González-Martín, and Ávila-Ortiz 2023). On the other hand, peri-implant mucositis was strongly correlated with a decrease in KTW. One risk factor associated with the severity of peri-implant mucositis is a decrease in the breadth of the KG surrounding dental implants (Grischke et al. 2019). Recent longitudinal data have further emphasized the importance of peri-implant phenotypic traits in sustaining peri-implant health, identifying thin soft tissues and increasing plaque buildup as important factors of peri-implant mucositis (Sabri et al. 2026). In implants with a KTW ≥ 2 mm, KTW influenced both the development of experimental peri-implant mucositis (particularly at 21 days) and the resolution phase following plaque removal. Investigated experimentally induced and reversible mucositis lesions, whereas our study evaluated naturally occurring peri-implant mucositis in a cross-sectional setting (Schwarz et al. 2018). The presence of KG around dental implants has been associated with healthier peri-implant tissues and a lower risk of peri-implantitis. This suggests that KG alone may not be a strong predictor and supports the multifactorial nature of peri-implant health (Fathi et al. 2025). Peri-implantitis was reported in 4.2% of implants surrounded by KG, whereas this percentage increased to 25% in implants lacking KG. These findings indicate that the presence of KG, either at the time of implant placement or during supportive periodontal care, had a significant beneficial effect (Roccuzzo et al. 2025). Implants with insufficient KTW have a greater prevalence of peri-implantitis (24.1% vs. 17%) and peri-implant mucositis (46.6% vs. 34.1%) than those with sufficient KTW, and insufficient KTW is significantly associated with peri-implant diseases. The study concluded that a thin GP and insufficient KG may be significant risk factors for peri-implant diseases (Gharpure et al. 2021). Following mechanical therapy, compared with those with KTW, implants with narrow or absent KG had less favorable clinical outcomes at the 24-week follow-up. The KTW affected the clinical resolution of peri-implant mucositis, as treatment success was 23.9% lower in the NKM group, and the presence of nonkeratinized mucosa decreased the likelihood of disease resolution by more than 80%. It has been concluded that sufficient KG may play an important role not only in maintaining peri-implant tissue health but also in the success of peri-implant mucositis treatment (Blasi et al. 2024). Conversely, Ravidà et al. (2021) reported that the quality of evidence supporting KG as a risk factor for peri-implant diseases, as well as the scientific basis for the commonly used 2-mm threshold in the literature, is quite low. It emphasized that this is not sufficient to consider KTW a strong and independent risk factor on its own and that higher-quality prospective studies are needed (Ravidà et al. 2022). No statistically significant correlation was observed between peri-implant mucositis or peri-implantitis and the buccal KTW. These findings suggest that a KT band may not be essential for maintaining peri-implant health (Oliveira et al. 2024). By contrast, after a 10-year follow-up period, the presence or absence of buccal keratinized mucosa had a statistically significant but small effect on peri-implant health (Mancini et al. 2024). Similarly, a recent multilevel regression analysis showed that the features of the soft tissues around the implant were independently related to peri-implant disease after adjustment for potential confounding factors. However, the study indicated stronger relationships for absence of KG than those identified in the present investigation (Isler et al. 2024). In our study, the association between increased GT and an elevated risk of peri-implant mucositis, along with the protective effect observed in the thin GP, partially contradicts the widespread assumption in the literature that thick KG is more stable and resilient. In contrast to earlier studies, peri-implant mucositis was related to larger GT in the present study. This finding may be explained by the cross-sectional design, which does not allow causal inference. Increased measured GT may have been due to inflammatory tissue swelling. Other confounding factors not assessed, or selection bias, may have affected the results. In addition, the small effect size (Cohen’s d = 0.27) indicates that although statistically significant, the therapeutic implications of this finding may be restricted.

Using standardized measurement methods, the present study provides a comprehensive evaluation of the relationships between soft tissue and various clinical periodontal parameters, which enhances the reliability of the findings. However, certain limitations should be considered. First, the cross-sectional design of the study precludes establishing causal relationships between gingival features and peri-implant health status. In particular, the increased GT observed at sites with mucositis may reflect inflammation-related edema rather than a pre-existing factor associated with mucositis. Second, clustering of implants may have compromised the independence of observations because numerous implants were included from certain patients. Moreover, intra-examiner calibration statistics were not provided, and measurement-related variability cannot be entirely excluded. Observed relationships may potentially reflect residual confounding due to unmeasured variables and possible selection bias. Another limitation of this study is that prosthetic characteristics that may influence peri-implant inflammation, including emergence profile angle, restoration contour, crown margin depth, restorative material, and cleanability, were not systematically recorded and therefore could not be included in the analysis. Finally, the results may not be generalizable, as this was a single-center study that excluded smokers.

## Conclusion

KTW did not differ significantly between the peri-implant health and mucositis groups. While GT and GP exhibited statistically significant differences, the small effect size indicates limited clinical significance. These results highlight the complexity of peri-implant tissue behavior and suggest that gingival characteristics alone may not adequately predict peri-implant disease. Clinicians should prioritize plaque control and regular supportive peri-implant maintenance rather than relying solely on KG dimensions when assessing peri-implant disease risk. Future well-designed longitudinal studies are warranted to clarify these relationships.

## Supplementary Information

Below is the link to the electronic supplementary material.

Supplementary Material 1

## Data Availability

The data that support the findings of this study are available from the corresponding author upon reasonable request.

## Abbreviations

KG: Keratinized gingiva
KTW: Keratinized tissue width
GT: Gingival thickness
GP: Gingival phenotype
PI: Plaque index
GI: Gingival index
PD: Probing depth
BOP: Bleeding on probing
GR: Gingival recession
CAL: Clinical attachment level
mPI: modified plaque index
