# Supplementary material for: Relationship between peri-implant soft tissue characteristics and peri-implant health and mucositis: a cross-sectional study
Source: Saudi Dent J. 2026 Oct 7;38(10):133. doi: 10.1007/s44445-026-00250-z (PMC13646208; doi:10.1007/s44445-026-00250-z)
Supplement: Supplementary file 1 [file 44445_2026_250_MOESM1_ESM.docx]

**Supplemantary Table 1**. Normality Tests Result

|  | Kolmogorov-Smirnov | | | Shapiro-Wilk | | | Skewness | Kurtosis |
| --- | --- | --- | --- | --- | --- | --- | --- | --- |
|  | Statistic | df | p | Statistic | df | Sig. |  |  |
| **PI** | 0.436 | 316 | 0.001 | 0.549 | 316 | 0.001 | 1.324 | 1.709 |
| **GI** | 0.242 | 316 | 0.001 | 0.800 | 316 | 0.001 | 0.718 | -0.932 |
| **PD** | 0.463 | 316 | 0.001 | 0.475 | 316 | 0.001 | -2.755 | 2.744 |
| **BOP (%)** | 0.200 | 316 | 0.001 | 0.822 | 316 | 0.001 | 0.690 | -0.888 |
| **GR** | 0.498 | 316 | 0.001 | 0.130 | 316 | 0.001 | 1.781 | 1.306 |
| **CAL** | 0.434 | 316 | 0.001 | 0.523 | 316 | 0.001 | -1.299 | 1.543 |
| **GT** | 0.372 | 316 | 0.001 | 0.705 | 316 | 0.001 | 0.669 | -0.348 |
| **KTW** | 0.160 | 316 | 0.001 | 0.869 | 316 | 0.001 | 1.150 | 1.370 |
